# Evaluation of selected organic fertilizers on conditioning soil health of smallholder households in Karagwe, Northwestern Tanzania

**DOI:** 10.1016/j.heliyon.2024.e26059

**Published:** 2024-02-08

**Authors:** Baraka Ernest, Amna Eltigani, Pius Z. Yanda, Anders Hansson, Mathias Fridahl

**Affiliations:** a Department of Medical Botany, Plant Breeding, and Agronomy, Muhimbili University of Health and Allied Sciences, P.O Box 65001, Dar es Salaam, Tanzania; b Programme Area “Next-Generation Horticultural Systems”-HORTSYS, Leibniz Institute of Vegetable and Ornamental Crops (IGZ), DE-14979, Großbeeren, Germany; c Institute of Resource Assessment, University of Dar es Salaam, P.O Box 35097, Dar es Salaam, Tanzania; d Department of Thematic Studies: Environmental Change, Centre for Climate Science and Policy Research (CSPR), Linköping University, SE-58183, Linköping, Sweden

**Keywords:** Organic fertilizers, Soil health, Cation exchange capacity, Soil organic carbon, Macronutrient, Micronutrient

## Abstract

Soil management is a strategy for improving soil suffering from problems such as low pH, nutrient deficiency, and erosion. The study evaluated the effects of human urine (HU), biogas slurry (BS), standard compost (StC), animal manure (AM), and synthetic fertilizer (SF) in comparison with no soil fertility management (NFM) on soil pH, cation exchange capacity (CEC), soil organic carbon (SOC), soil moisture content, nitrogen (N), phosphorus (P), potassium (K), calcium (Ca), magnesium (Mg), sodium (Na), copper (Cu), zinc (Zn), manganese (Mn), and iron (Fe) in the Karagwe district, a Northwestern Tanzania. Four household farms representing each soil amendment type were selected for soil sampling. A total of 192 soil samples were collected and air-dried. After laboratory analysis, BS-enriched soil had the highest pH (6.558), CEC (23.945 cmol+/kg), SOC (5.573%), soil moisture (5.573%), N (0.497%), P (247.130 mg/kg), K (3.036 cmol+/kg), Ca (18.983 cmol+/kg), Mg (4.076 cmol+/kg), Na (2.960 cmol+/kg), and Cu (12.548 mg/kg). Similar soil properties were lower in NFM than in the other soils. The soil properties on the chosen farms did not differ significantly depending on the sampling zone for each organic fertilizer. Therefore, the result indicates that all evaluated organic fertilizers improved soil health compared to NFM, but BS and HU fertilizers led to relatively better soil health improvements than StC, AM, and SF.

## 1 Introduction

Soil health refers to the ability of soil to function as a living ecosystem that supports the health of humans, animals, and plants [1]. The health of the soil is evaluated based on its chemical, physical, and biological characteristics [2]. Globally, healthy agricultural ecosystems, which encompass most productive soils, occupy approximately 11% of the Earth's land surface [3]. However, anthropogenic activities have led to the highly degraded state of nearly 25% of the world's productive soil resources, with an additional 44% classified as moderately degraded [4]. Sub-Saharan Africa (SSA) in particular suffers from a high frequency of severely degraded soils, with around 65% of the agricultural area falling into this category [5]. Consequently, jeopardizing the livelihoods of more than 65% of the population in SSA who rely on agriculture [6].

Similar to several parts of SSA, the agricultural soils in Karagwe, Tanzania, are experiencing issues such as erosion, reduced water content, nutrient loss [7], and acidity (pH < 4.0) [8]. The dominant soil type in Karagwe, Andosol, suffers from low phosphorus (P) deficiency due to its high ability for P-fixation [8]. These challenges are exacerbated by continuous ploughing, with farmers adding little or no organic fertilizers back to the soil [[9], [10], [11], [12], [13]]. Consequently, the soil system based on bananas is at risk due to deteriorating soil health. Therefore, regular application of organic fertilizers is necessary to sustainably improve soil health [14,15].

Organic fertilizer refers to naturally occurring organic materials that contain sufficient nutrients to enhance soil health [16,17]. Through the provision of a variety of soil nutrients, organic fertilization techniques offer an efficient means of sustainably preserving soil health over time [15,18,19]. Several studies have demonstrated that adding organic fertilizers to the soil alters the soil's organic matter (SOM), acidity, and cation exchange capacity (CEC), while also increasing soil moisture content [15,18,20]. Increasing the amount of SOM enhances the level of soil organic carbon (SOC), which is a vital precursor to essential and trace elements [21]. Therefore, the use of organic fertilizers enhances the resilience of degraded soils, promoting long-term soil health sustainability [22].

In the Karagwe district, approximately 78% of farmers use organic fertilizers, while less than 1% of cultivated land relies on synthetic fertilizers (SF) [8]. The main locally accessible organic materials in the Karagwe district, used as organic fertilizers, include standard compost (StC) made from crop leftovers, kitchen scraps, food scraps, animal bones, and cooking ash; and animal manure (AM) [8]. These organic fertilizers, are locally made and applied in agricultural fields for soil health improvement [23]. Organic fertilizers are safer than chemical fertilizers, despite their slower release of nutrients, lower levels of essential soil nutrients, and restricted availability of organic matter sources [[24], [25], [26], [27]].

Due to these challenges, in 2008, local non-governmental organizations MAVUNO^1^ and CHEMA^2^ collaborated with Engineers Without Borders^3^ from Germany to launch initiatives aimed at helping small household farmers improve the quality of their soil [8]. These initiatives offer an integrated approach to resource management by utilizing locally available resources and recycling nutrients [8]. One specific method is the recycling of by-products, such as sterilized human urine (HU) and biogas slurry (BS), back into the agroecosystem for managing soil health. According to World Health Organization [28], urine has been deemed safe for use as an organic fertilizer in agriculture. Therefore, many households in the Karagwe district have adopted the use of HU and BS as organic fertilizers for their fields [29,30].

As demonstrated by a series of long-term experiments, organic fertilizer application has been shown to improve soil health in agroecosystems [[31], [32], [33]]. Consequently, smallholder farmers have adopted the use organic fertilizers to their farms in order to maintain soil health. However, there is a lack of studies comparing the contributions of different organic fertilizers in effectively conditioning soil health over time in smallholder farmlands. Currently, there is limited information available on the ability of locally produced organic fertilizers, recycled to restore the health of degraded soil.

Therefore, the specific objective of this study was to evaluate the most effective organic fertilizer in improving health (soil pH, cation exchange capacity (CEC), soil organic carbon (SOC), soil moisture content, nitrogen (N), phosphorus (P), potassium (K), calcium (Ca), magnesium (Mg), sodium (Na), copper (Cu), zinc (Zn), manganese (Mn), and iron (Fe)) of smallholder farmlands in Karagwe. The study employed smallholder farmlands applied with human urine (HU), biogas slurry (BS), standard compost (StC), animal manure (AM), and synthetic fertilizer (SF) in comparison with no soil fertility management (NFM). We hypothesize that, quantifying the relative importance of various types of organic fertilizers may prove beneficial to sustain soil health of smallholder farmlands over time.

## 2 Materials and methods

### 2.1 Description of the study area

**Fig. 1:**
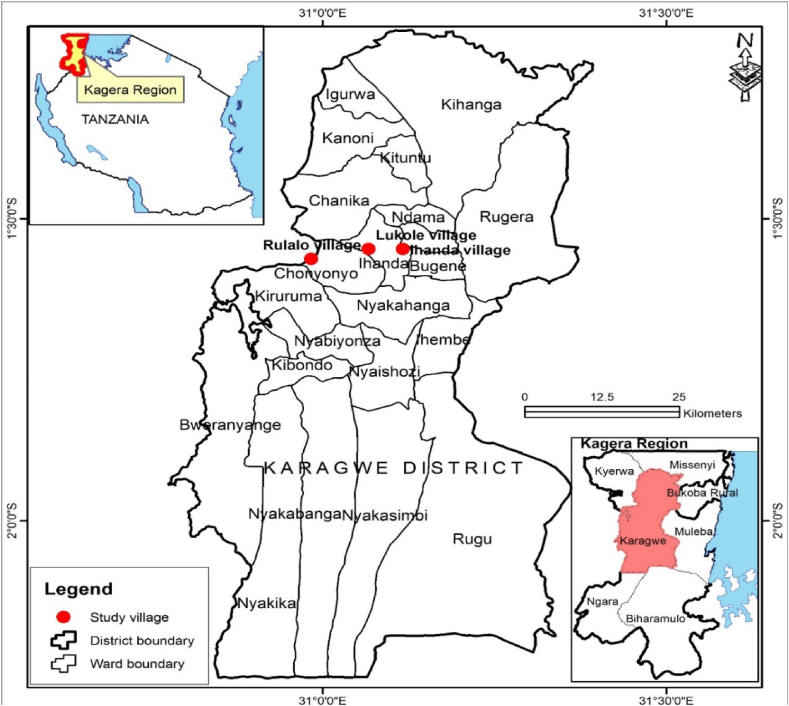
Location of the study area.

The investigation of the study took place in the Karagwe District of Northwestern Tanzania, which is located within the Kagera Region. Specifically, the study focused on the villages of Ihanda, Lukole, and Rulalo with geographical coordinates 1°34′22″S and 31°3′29″E; 1°33′4″S and 31°4′56″E; and 1°34′38″S and 30°59′46″E respectively (Fig. 1). Engineers without Borders collaborated with smallholder farmers in these areas through the BiogaST, EfCoiTa, and CaSa projects. These projects aim to integrate nutrient and carbon recycling methods. The organic fertilizer program, initiated by MAVUNO and CHEMA, was implemented in these study areas.

The district has an average annual temperature of 26 °C, indicating a tropical highland climate. The rainfall is bimodal, with peaks occurring from March to May and from September to December [34]. Due to this climate, most annual crops can be harvested twice a year [35]. Home farms in the area grow trees, vegetables, coffee, and bananas. The predominant soil type is andosol, which forms from the weathering of volcanic materials in higher altitudes and volcanic areas [36]. The soil in the area has a pH of 3.8–4.2, low nutrient availability (especially P), and is prone to soil erosion due to the hilly terrain [8].

### 2.2 Soil sampling procedures

**Fig. 2:**
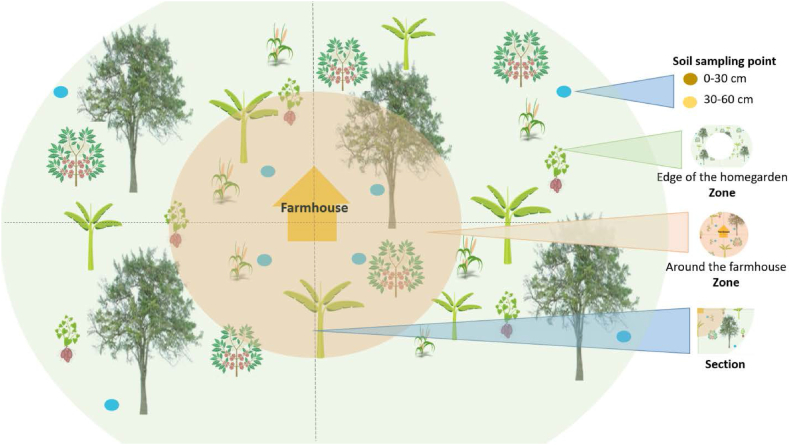
Illustration of the soil sampling scheme at the household farm at a depth of 0–30 cm. The sampling covers the four quarters (sections) of the field and two zones (near the household farm and at the edge of the household farm far from the household farm).

Household farms of smallholder farmers, who were participating in capacity-building program organized by MAVUNO for local farmers on soil fertility management, were selected for this study. The study aimed to compare soil samples from farms that had applied different organic fertilizers, namely HU, BS, StC, AM and SF, control group NFM. To gather the samples, a purposeful sampling method was used to identify four household farms for each type of organic fertilizer. The farms were divided into four sections, representing each quarter of the farm, in two zones: near the household farm and at the farm's edge, far from the household farm (Fig. 2). At each section, eight soil samples were randomly taken at points using a soil auger. The samples were collected at a soil depth of 0–30 cm. Soil nutrient balances in smallholder agriculture are typically positive near the household farm but often become negative as one moves farther away. To account for this, two zones within the household farm were sampled: near the farm and at its periphery. The area surrounding household farms, being in close proximity to the house, often receives sufficient inputs of agricultural and domestic residues [29,37]. After being sent to the lab for soil moisture content assessment, 192 soil samples were air-dried. These samples were then used to measure pH, cation exchange capacity (CEC), soil organic carbon (SOC), nitrogen (N), potassium (K), available phosphorus (P), exchangeable calcium (Ca), magnesium (Mg), sodium (Na), as well as various metals such as copper (Cu), zinc (Zn), manganese (Mn), and iron (Fe).

### 2.3 Determination methods of selected soil health properties

A pH meter was used to measure the water suspension in a 1:2.5 soil [38]. CEC was calculated using a flame photometer and an ammonium acetate extraction method buffered at pH 7 [39]. The amount of SOC was determined using the dichromate oxidation method [40]. After the soil samples were brought to the lab and dried in an oven at 105 °C for 48 h to reach a constant weight, the soil moisture contents were determined using the gravimetric moisture estimation method [41]. The semi-micro Kjeldahl method, which involves titration and distillation steps, was used to determine N [42]. Olsen's method was used to calculate available P in sodium bicarbonate (NaHCO3) [43]. The available K, Ca, and Mg were tested using the atomic absorption spectrophotometric method [44]. Exchangeable bases Ca, Mg, Na, and K were extracted using a standard ammonium acetate solution buffered at pH 7.0. The readings for Ca and Mg were determined using the atomic absorption spectrophotometric method, while Na and K were determined using a flame photometer [45]. Cu, Zn, Mn, and Fe availability were assessed using an atomic absorption spectrophotometric technique.

### 2.4 Statistical analysis

The study used a one-way analysis of variance to analyze the average differences in selected soil health parameters among the five organic fertilizers and NFM. A parametric Gaussian normal distribution was considered when testing the data. The Tukey test was employed to evaluate the variations between the means of organic fertilizer for each selected soil health parameter at a probability level of *p* ≤ 0.05.

## 3 Results

### 3.1 Effect of organic fertilizers on soil pH

The pH of the soil in the selected household farms is significantly affected by the use of organic fertilizers. Among the household farms, there was a significant (*p* ≤ 0.05) variation in soil pH depending on the type of organic fertilizer used (Table 1). The pH of the soil was highest in household farms treated with BS, followed by household farms amended with HU, and lowest in household farms with NFM. According to Wang et al. [46], the use of organic fertilizers is primarily responsible for the observed change in soil pH among household farms. However, there were no significant variations in soil pH observed in soils amended with HU and AM, AM and SF, StC and the NFM control group (Table 1). No significant differences (*p* > 0.05) in soil pH were found for any type of organic fertilizer in Z1 (i.e., the area close to the center of the household farm) or Z2 (i.e., the area at the edge of the household farm, far from the center) (Table 2).

**Table 1:** Selected soil chemical properties of each household farm per organic fertilizer for soil sampled 0–30 cm depth.

Organic fertilizer	%			cmol+/kg			
	SOC	Moisture content	Soil pH	CEC	Ca	Mg	Na
**HU**	3.762d	2.564d	6.302b	22.058b	17.703a	3.077b	1.219b
**BS**	5.573a	4.772a	6.558a	23.945a	18.983a	4.076a	2.960a
**StC**	4.382c	4.125c	5.072d	17.060d	14.121 cb	2.058d	0.630cd
**AM**	4.784b	4.544b	6.101bc	18.037c	15.078b	2.488c	0.801c
**SF**	4.723b	2.361d	5.949c	17.022d	14.660b	2.097d	0.653cd
**NFM**	3.336e	1.729e	4.997d	16.828d	10.852d	1.155e	0.361d

HU:Human urine; BS:Biogas slurry; StC:Standard compost; AM:Animal manure; SF:Synthetic fertilizer; NFM:without fertility management; SOC:Soil organic carbon; CEC:Cation exchange capacity; Ca:calcium; Mg:magnesium; Na:sodium.

*Means in the same column followed by the same alphabets are not significantly different using Tukey posthoc test at *p* ≤ 0.05.

**Table 2:** Selected soil chemical properties near the center of the household farm (Z1) and at the edge of the household farm far from the center of the household farm (Z2) of the household farm per organic fertilizer for soil sampled 0–30 cm depth.

Organic fertilizer	Zonation	%			cmol+/kg			
		OC	Moisture content	Soil pH	CEC	Ca	Mg	Na
**HU**	**HU-Z1**	3.763e	2.591de	6.251bc	22.366b	17.870b	3.124bc	1.260bc
	**HU-Z2**	3.761e	2.537de	6.352bc	21.751b	17.536b	3.030bc	1.179bc
**BS**	**BS-Z1**	5.553a	4.887 ab	6.577 ab	24.056a	19.111 ab	4.098a	3.124a
	**BS-Z2**	5.594a	4.657 ab	6.539 ab	23.834a	18.855 ab	4.053a	2.796a
**StC**	**StC-Z1**	4.341 db	4.153c	5.009ef	17.149ec	14.291ec	2.136de	0.651ebf
	**StC-Z2**	4.423 db	4.098c	5.134ef	16.972ec	13.951ec	1.979de	0.609ebf
**AM**	**AM-Z1**	4.858bc	4.546b	6.073cd	18.187cd	15.151cd	2.476cd	0.806ced
	**AM-Z2**	4.711bc	4.542b	6.128cd	17.887cd	15.005cd	2.500cd	0.797ced
**SF**	**SF-Z1**	4.763cd	2.388e	5.889d	17.208def	14.869de	2.060ec	0.694 db
	**SF-Z2**	4.683cd	2.334e	6.009 db	16.836def	14.451de	2.134ec	0.613 db
**NFM**	**NFM-Z1**	3.366f	1.709f	4.918f	16.823fc	10.748f	1.209f	0.396 fcd
	**NFM-Z2**	3.306f	1.748f	5.076f	16.832fc	10.955f	1.101f	0.322 fcd

HU: Human urine; BS: Biogas slurry; StC: Standard compost; AM: Animal manure; SF: Synthetic fertilizer; NFM: Without fertility management; HU-Z1: Human urine near the center of the household farm; HU-Z2: Human urine at the edge of the household farm far from the center of the household farm; BS-Z1: Biogas slurry near the center of the household farm; BS-Z2: Biogas slurry at the edge of the household farm far from the center of the household farm; StC-Z1: Standard compost near the center of the household farm; StC-Z2: Standard compost at the edge of the household farm far from the center of the household farm; AM-Z1: Animal manure near the center of the household farm; AM-Z2: Animal manure at the edge of the household farm far from the center of the household farm; SF-Z1: Synthetic fertilizer near the center of the household farm; SF-Z2: Synthetic fertilizer at the edge of the household farm far from the center of the household farm; NFM-Z1: Without fertility management near the center of the household farm; NFM-Z2: Without fertility management at the edge of the household farm far from the center of the household farm; OC: Organic carbon; CEC: Cation exchange capacity; Ca: calcium; Mg: magnesium; Na: sodium.

*Means in the same column followed by the same alphabets are not significantly different using Tukey posthoc test at p ≤ 0.05.

### 3.2 Effect of organic fertilizers on CEC

The results indicate that the CEC of household farms treated with different types organic fertilizers varied significantly (*p* < 0.05) (Table 1). The highest soil CEC was found on household farms treated with BS, while the lowest was found on NFM farms. Additionally, the highest soil CEC was observed on HU-treated household farms. The addition of organic fertilizer to household farms is thought to be the cause of the higher CEC in the soils [18,47]. However, Table 1 shows that there were no significant variations in soil CEC between household farms treated with StC and SF, or between household farms treated with StC and NFM and farms treated with SF and NFM (*p* > 0.05). Furthermore, there were no significant differences in CEC between Z1 and Z2 of the household farms for any type of organic fertilizer on the selected household farms (Table 2).

### 3.3 Effect of organic fertilizers on SOC

The soil's SOC content, when treated with different organic fertilizers, exhibited significant differences (*p* ≤ 0.05). According to Table 1, household farms applied with BS had the highest SOC, followed by AM-applied household farms, while household farms applied with NFM had the lowest SOC. However, the increased CEC according to Zhou et al. [18] is a result of incorporating organic fertilizers to household farms. The SOC content of soils applied with SF and AM did not show any noticeable difference (*p* > 0.05) (Table 1). Additionally, there were no significant (*p* > 0.05) variations in SOC content between samples from Z1 and Z2 for any type of organic fertilizer used on the selected household farms (Table 2).

### 3.4 Effect of organic fertilizers on soil moisture content

Based on the type of organic fertilizers used, the data show significant differences (*p* < 0.05) in the soil moisture contents among the household farms. Table 1 reveals that soil of the household farms applied with AM had the lowest moisture content, while household farms applied with BS had the highest moisture content. The improvement in the moisture contents of household farms is primarily attributed to organic fertilizers [48]. On the other hand, NFM had the lowest moisture content. However, Table 1 did not indicate any significant differences (*p* > 0.05) between HU and SF. The zoning data in Table 2 demonstrate that there were no significant differences (*p* > 0.05) in the soil moisture contents between Z1 and Z2 of the household farms for each type of organic fertilizer.

### 3.5 Effect of organic fertilizers on soil macronutrients

**Fig. 3:**
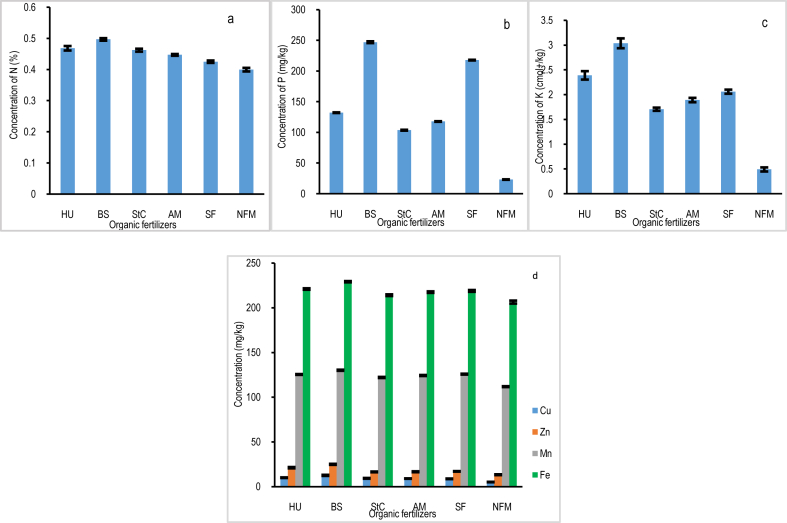
Concentration in the soil of (a) N: nitrogen (%); (b) P: phosphorus (mg/kg); (c) K: potassium (cmol+/kg); (d) Cu: copper (mg/kg); Zn: zinc (mg/kg); Mn: manganese (mg/kg); and Fe: iron (mg/kg) per household farm per organic fertilizer.

**Fig. 4:**
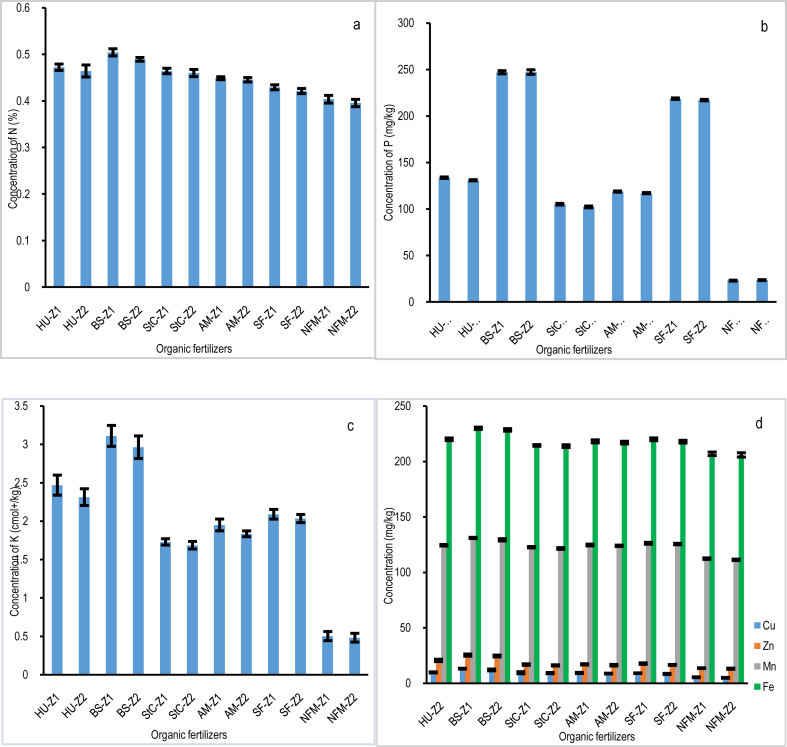
The concentration of (a) N: nitrogen (%); (b) P: phosphorus (mg/kg); (c) K: potassium (cmol+/kg); (d) Cu: copper (mg/kg); Zn: zinc (mg/kg); Mn: manganese (mg/kg); Fe: iron (mg/kg) near the center of the household farm and at the edge of the household farm far from the center of the household farm.

The macronutrient status of household farms varied significantly among organic fertilizers. Depending on the type of organic fertilizer, there were significant differences (*p* < 0.05) in the concentrations of plant macronutrients among the household farm soils. The household farms applied with BS had the highest levels of N, P, K (Fig. 3a, b, c), as well as Ca, Mg, and Na (Table 1). On the other hand, the NFM household farms had the lowest levels of N, P, K (Fig. 3a, b, c), Ca, Mg, and Na (Table 1). Zhou et al. [18] reported a significant change in the amount of macronutrients following the application of organic fertilizers. However, the zonation data show that there were no statistically significant differences (*p* > 0.05) in N, P, K (Fig. 4a, b, c), Ca, Mg, and Na (Table 2) between soil samples from Z1 and Z2 of the household farm for any type of organic fertilizer used in the selected household farms.

### 3.6 Effect of organic fertilizers on soil micronutrients

Despite micronutrients being necessary in small amounts, their absence can have a significant impact on soil health. The micronutrient data for the household farms applied with various types of organic fertilizers showed statistically significant differences (*p* < 0.05) in Cu, Zn, Mn, and Fe. Household farms applied with BS had the highest concentrations of Cu, Zn, Mn, and Fe, followed by household farm soils applied with HU. In contrast, NFM household farms had the lowest concentrations of micronutrients (Fig. 3d). Micronutrient levels have changed as a result of adding organic fertilizers, which are considered the best source of micronutrients for the household farms [49]. According to the zonation data, there were no significant differences (*p* > 0.05) in the concentrations of Cu, Zn, Mn, and Fe between Z1 and Z2 of the household farm for any type of organic fertilizer on the chosen household farms (Fig. 4d).

## 4 Discussion

Using various organic fertilizers, our findings demonstrated the distinct effects of pH levels on household farms. All of the soil samples exhibit acidity, as evidenced by the pH values (Table 1), which range from 4.997 to 6.558. According to Oyedele et al. [50], this indicates that the soil contains a significant amount of exchangeable acidity (H^+^ + Al^3+^), accounting for the majority of the soil's acidity. The acidic nature of the soil in the area, coupled with the high acidity observed in NFM (pH 4.997) and SF (pH 5.949) household farms, may explain the improper application of inorganic fertilizers and the absence of management practices. The combination of soil acidity and continuous use of inorganic fertilizers without a liming program leads to high soil acidity in agricultural soils [50]. Furthermore, Berek & Hue [51], Rusli et al. [52], and Smider & Singh [53] discovered that basic cations such as calcium (Ca^2+^), magnesium (Mg^2+^), and potassium (K^+^) can dissolve in water as oxides or carbonates and produce hydroxyl (OH^−^), which raises the pH of the soil. This study corroborates these findings by demonstrating that organic fertilizers in household farms receiving BS have a positive and more effective impact on the level of basic cations (Table 1). Chang-ai et al. [54] have also provided support for the promising results of boosting soil pH with BS, showing that it can effectively raise the pH of acidic soil. A study conducted in Karagwe found that low soil pH, ranging between 3.8 and 4.2 in agricultural fields, is one of the factors that limit agricultural yield [8]. According to a study by Bach et al. [55], recycling materials from BS can solve low pH issues in soil when applied to acidic soils.

The results of Niyungeko et al. [56], who reported comparable outcomes of the application of BS in soil, are consistent with the observed increase in CEC in household farms after BS application. The average CEC of 23.945 cmol+/kg in household farms amended with BS is primarily due to the correspondingly higher soil pH that results from applying BS to the household farms (Table 1). This value is comparable to that of soils amended with HU, StC, AM, SF, and NFM. Aprile & Lorandi [57], Bache [58], and Gul et al. [59] provide compelling evidence supporting the argument that CEC is highly dependent on soil pH. Table 1 shows that household farms receiving HU have a CEC of 22.058 cmol+/kg. On the other hand, households receiving BS demonstrate positive outcomes in terms of increasing CEC for improving soil health through the catalyzation of exchangeable bases. Previous research by Debebe & Itana [60] and Kumar et al. [61] supports the idea that adding BS to the soil enhances and raises CEC. Additionally, CEC was found to be higher near the center of the household farm compared to its periphery, which is further away (Table 2). Among the household farms, the CEC was higher in the vicinity of the BS household farm's center compared to the corresponding CEC at the household farm's periphery, which is further from the center (Table 2). This result supports the findings of Krause [37] and Reetsch et al. [29], who noted that the household farm, being surrounded by the house, receives frequent inputs of domestic and agricultural residues. These inputs regulate the availability of soil nutrients for agricultural purposes.

Research indicates that organic fertilizers affect the amount of nutrients in the soil that are easily accessible for plant development and growth [62,63]. According to the results (Fig. 3a, b, c, and Table 1), organic fertilizers such as HU, BS, StC, and AM had a greater positive impact on soil nutrients than SF and NFM. When compared to other types of organic fertilizers, BS-amended household farms have the highest concentration of soil nutrients (Fig. 3a, b, c, and Table 1), followed by HU. The findings of this study are in line with Kumar et al. [64] suggesting that organic fertilizers, specifically BS, carry macro and micronutrients more effectively. Further evidence show that BS are rich in mineral nutrients such as nitrogen (N), phosphorus (P), potassium (K), calcium (Ca), magnesium (Mg), sodium (Na), copper (Cu), zinc (Zn), manganese (Mn), iron (Fe), and sulfur (S) [61,[65], [66], [67]]. These nutrients are more important for soil fertilization and soil health improvement. Additionally, BS fertilizer has been shown to have a greater impact on the availability of both macro and micronutrients in the soil due to its influence on soil pH and CEC when compared to other types of organic fertilizers (Table 1). The results investigated by Chang-ai et al. [54], Ng et al. [68], and Niyungeko et al. [56] provide support for the findings of this study by showing that BS raises soil pH and CEC, increasing the availability of macro- and micronutrients for plant growth and development. As predicted, NFM household farms have the lowest pH, CEC, and concentrations of macro- and micronutrients (Table 1 and Fig. 3a, b, c, d). These results suggest that in order to overcome the limitations of soil infertility and establish healthy and fertile soils for crop production, NFM household farms must apply their soil with appropriate organic fertilizer to counterbalance soil nutrient depletion [68]. However, when compared to the peripheries of the household farm, which is located far from the center, the majority of macro and micronutrients were slightly higher in proximity to the center (Fig. 4a–c, d, and Table 2). Consequently, BS presents itself as a more affordable and safer option than SF for nutrient supply, making it a potential soil conditioner and fertilizer.

Several agricultural practices have been found to enhance SOC storage. These practices include adding AM, incorporating plant residues, growing and green manures like cover crops in agricultural fields [[69], [70], [71], [72], [73], [74]]. The findings support the results of this study, which show that HU, BS, StC, AM, and SF all contribute to the significant amount of SOC in the soil. The household farms that received BS had considerably higher concentrations of SOC, followed by soils applied with AM. On the other hand, the soils of NFM household farms had the lowest concentration of SOC (Table 1). According to Koishi et al. [75], the distribution of SOC in various fractions was impacted by organic fertilizers. These results are consistent with a number of scientific studies showing that a wide variety of organic fertilizers, such as manure and compost, increases a significant amount of SOC [[76], [77], [78]]. BS adds the necessary SOC to improve soil fertility compared to other organic fertilizers. However, investigation by Koishi et al. [75] show that soils applied with organic fertilizers revealed higher concentration of SOC, while soils applied with SF showed lower concentration of SOC. Among the various types of organic fertilizers tested in this study, BS showed the highest amount of SOC storage, indicating promising results of conserving soil carbon over time. This favorable outcome results in SOC being firmly bound, stable, strongly protected, and degrading more slowly [79,80]. A study by Cotrufo et al. [81] also found that stable SOC promotes carbon sequestration and increases soil fertility due to its slower carbon turnover. Increased binding agents like humic acid and iron-aluminum oxides in SOC are responsible for the stability of SOC after BS application [82,83]. This increased stability of the soil helps it withstand external disturbances and reduces the rate of turnover of SOC [84]. By receiving BS as organic fertilizer, farmers can utilize the fertilizer to stabilize SOC to highlight its importance in the global carbon cycle. This could be an excellent soil management strategy to increase SOC in agroecosystems, offering a promising method of mitigating the effects of climate change and helping to achieve other sustainable development goals (SDGs), such as SDG2 (zero hunger) and SDG15 (life on land) [75,85].

According to Subhan et al. [86], using organic byproducts has positive effects on soils by enhancing the physical, chemical, and biological qualities of the soil. A study by Haque et al. [87] found that organic materials from different sources, when used as soil fertilizers, can increase soil moisture storage and nutrient enrichment. Similar results were observed in this study, where HU, BS, StC, AM, and SF significantly increased soil moisture contents compared to NFM (Table 1). Haque et al. [87] also stated that applying various organic fertilizers to soil has the potential to greatly improve soil moisture retention and provide plants with essential nutrients. The results of this study revealed that household farms applied with BS had the highest soil moisture content at 5.573%, followed by AM at 4.784% and SF at 4.723%. The lowest soil moisture content was found in NFM at 3.336% (Table 1). Except for NFM-Z1, the data near the household farm showed the highest soil moisture contents across all types of organic fertilizers (Table 2). BS-Z1 exhibited the highest soil moisture content. These findings align with a study by Ye et al. [88], which demonstrated that BS organic fertilization improved soil moisture content more effectively than SF, StC, and AM. Similar research has shown that the use of organic fertilizer enhances crop water use efficiency and soil moisture content [88]. Due to climate change, there is a possibility of increased and sustained soil moisture during periods of high evaporation and unpredictable precipitation [89]. In such circumstances, incorporating BS into the soil can enhance its ability to retain moisture over time.

## 5 Conclusion

Organic fertilizers are considered to enhance soil health for sustainable agriculture. When compared to soils applied with human urine (HU), standard compost (StC), animal manure (AM), and synthetic fertilizer (SF), the addition of biogas slurry (BS) to soils significantly improved selected soil health parameters. These include soil pH, cation exchange capacity (CEC), soil organic carbon (SOC), soil moisture content, nitrogen (N), phosphorus (P), potassium (K), exchangeable calcium (Ca), magnesium (Mg), sodium (Na), copper (Cu), zinc (Zn), manganese (Mn), and iron (Fe). It is worth noting that, compared to household farms without fertilizer management (NFM), almost every type of organic fertilizer examined in this study showed improvements in the selected soil health parameters. The soil health parameters for each type of organic fertilizer on the selected household farms were higher near the center of the household farm compared to the periphery, which was farther away from the center. The availability of plant macro- and micronutrients in the soil is directly influenced by the independent soil parameters, soil pH, and CEC. Due to its higher soil pH and CEC, BS can provide a significant amount of macro and micronutrients. Additionally, BS has shown its ability to retain SOC, making it a potential tool for mitigating climate change by storing carbon in soils. Restoring a significant amount of moisture in the soil, which plants can utilize during periods of water stress, is made possible by BS to soils. Moreover, BS has proven to be a successful organic fertilizer, effectively reducing the reliance on chemical fertilizers. Therefore, this study recommends the implementation of BS in small-scale farming operations as it offers farmers a valuable means of reducing the fertilizer burden and improving field and environmental sustainability. Additionally, apart from being more effective than NFM, other types of organic fertilizers can also be suggested.

## Data availability statement

Data will be made available on request.

## CRediT authorship contribution statement

**Baraka Ernest:** Writing – original draft, Methodology, Investigation, Formal analysis, Data curation, Conceptualization. **Amna Eltigani:** Writing – original draft, Methodology, Investigation, Formal analysis, Data curation, Conceptualization. **Pius Z. Yanda:** Writing – review & editing, Supervision, Investigation, Conceptualization. **Anders Hansson:** Writing – review & editing, Supervision, Project administration, Funding acquisition. **Mathias Fridahl:** Writing – review & editing, Supervision.

## Declaration of competing interest

The authors declare that they have no known competing financial interests or personal relationships that could have appeared to influence the work reported in this paper.

## Footnotes

1 MAVUNO: Non-profit NGO and is based in Karagwe district, Kagera region, Tanzania. The NGO aims to foster self-determined development and rural empowerment through working in close collaboration with community members.

2 CHEMA: Non-profit NGO based in Karagwe district, Kagera region, Tanzania. CHEMA aims to improve rural livelihoods through comprehensive village-based natural resource management and sustainable agriculture.

3 Engineers Without Borders: An international solidarity organization for engineering and technology. It focuses on both education and the implementation of sustainable engineering projects at the grass-root level.
